# Factor Structure and Convergent Validity of the Short Version of the Bielefeld Partnership Expectations Questionnaire in Patients With Anxiety Disorder and Healthy Controls

**DOI:** 10.3389/fpsyg.2022.638644

**Published:** 2022-03-09

**Authors:** Uwe Altmann, Katja Brenk-Franz, Bernhard Strauss, Katja Petrowski

**Affiliations:** ^1^Institute of Psychosocial Medicine, Psychotherapy and Psycho-oncology, University Hospital Jena, Jena, Germany; ^2^Clinic and Polyclinic for Psychotherapy and Psychosomatics, Technische Universität Dresden, Dresden, Germany; ^3^Institute of Medical Psychology and Medical Sociology, Johannes Gutenberg-Universität Mainz, Mainz, Germany

**Keywords:** adult attachment, self-rating, anxiety disorder, factor analysis, scales, relationship

## Abstract

The short version of the Bielefeld Partnership Expectations Questionnaire (BPEQ-12) assesses the partner-related attachment dimensions fear of rejection, readiness for self-disclosure, and conscious need for care. The presented study investigated the factor structure in two samples and evaluated the convergent validity of scales. The sample included *N* = 175 patients with panic disorder and/or agoraphobia and *N* = 143 healthy controls. Besides, the BPEQ, the Experiences in Close Relationships Questionnaire (ECR), and the Brief Symptom Inventory (BSI) were assessed as well, and the Adult Attachment Prototype Rating (AAPR) was conducted. A confirmatory factor analysis of the three factor model (using a WLSMV estimator) revealed an acceptable model fit for the entire sample, patients and controls in terms of low RMSEA and SRMR (< 0.08) and high CFI and TLI (> 0.95). We found metric, scalar, and strict measurement invariance for the presence of anxiety disorder (ΔCFI ≤ –0.01 and ΔRMSEA ≥ 0.01). However, only for fear of rejection and readiness for self-disclosure the reliability was acceptable (Cronbach’s α > 0.7), and convergent validity in terms of large correlations (*r* > 0.7) with the ECR scales was found in both samples. The scale conscious need for care had a questionable reliability (Cronbach’s α > 0.6) and correlated only slightly with ECR-R scales. We conclude that fear of rejection and readiness for self-disclosure of the BPEQ-12 are reliable and valid scales for measuring partner-related attachment in healthy and clinical samples.

## Background

The concept of the internal working model (Bowlby, 1973, 1983) is of central importance for the theory of adult attachment. Research has shown that the development of attachment-specific internal working models of the self and others influence emotion regulation and interpersonal patterns in childhood as well as later experiences in close relationships (Mikulincer et al., 2003). A further milestone in attachment research is the typology of the secure, avoidant, and anxious-ambivalent attachment style by Ainsworth et al. (2015).

A highly discussed topic is the measurement of adult attachment (Garbarino, 1998; Roisman et al., 2007; Jewell et al., 2019), which can be divided into classifications and dimensional models (Fraley et al., 2015). Furthermore, the instruments differ in regard to the focus on the respective attachment figure (mother, father, current partner) and type of assessment (self-ratings, semi-protective methods, or expert ratings based on interviews). However, the different attachment instruments have no convergent validity or moderate at best (Shaver et al., 2000; Roisman et al., 2007; Jewell et al., 2019; Strauss et al., 2022). It is assumed that convergent validity is higher, the more the two instruments match regarding method, domain, and concept (Bartholomew and Shaver, 1998).

The Bielefeld Partnership Expectations Questionnaire (BPEQ; Höger and Buschkämper, 2002; Höger et al., 2008; Pollak et al., 2008) is a self-rating of partner-related attachment dimensions. With 30 items, the scales “fear of rejection,” “readiness for self-disclosure,” and “conscious need for care” are assessed reliably (Cronbach’s α = 0.79-0.95; Kirchmann et al., 2007; Höger et al., 2008; Petrowski et al., 2010). The items are based on the Bielefeld Client Expectations Questionnaire (BCEQ; Höger, 1999; Pollak et al., 2008) whereby the term “therapist” is often replaced by the term “partner.” The items of the BCEQ are self-descriptions and measure the patient’s expectations regarding the therapeutic relationship. However, the BPEQ is not limited to psychotherapy only, since the questionaire addresses the romantic/marital relationship.

In contrast to other attachment self-ratings, the BPEQ provides dimensional attachment measures and a classification into the attachment patterns secure, partially secure, (insecure) avoidant-withdrawing, (insecure) ambivalent-clinging, and (insecure) ambivalent-withdrawing (Höger and Buschkämper, 2002; Grau et al., 2003). Suggested by Collins and Read (1990) the classification algorithm considers the individual values of three scales (fear of rejection, readiness for self-disclosure, and conscious need for care) and maps them into a small number of attachment patterns based on cluster analyses (Höger, 1999; Pollak et al., 2008). According to Höger et al. (2008), the attachment patterns partially secure and avoidant-withdrawing can be merged to the so-called avoidant attachment pattern, and the ambivalent-clinging and ambivalent-withdrawing can be merged to the ambivalent attachment pattern.

Regarding the validity of the BPEQ scales, hypothesis-conform correlations were found with social support, relationship satisfaction (Petrowski et al., 2010), self-rated attachment anxiety, attachment avoidance (Grau et al., 2003) and attachment security ratings of the adult attachment prototype rating (Kirchmann et al., 2007), which is an interview-based expert rating of attachment (Strauss et al., 1999). The validity of the BPEQ attachment patterns is supported by the findings that the secure attachment pattern was found to be associated with positive parental rearing behavior and the insecure attachment pattern with negative experiences of parental rearing (Schumacher et al., 2004). Furthermore, as expected in a clinical sample of patients with eating disorders, the ambivalent attachment pattern was observed comparably often (Steins et al., 2002).

Recently, a short version of the BPEQ including 12 items (BPEQ-12, in German BFPE-12; Altmann et al., 2018) was developed using two different samples for exploratory factor analysis and confirmatory factor analysis. The reliability of the scales (Cronbach’s α > 0.799) and the concordance of the attachment patterns assessed with the orginal and the short version of the BPEQ were good (κ > 0.6) (Altmann et al., 2018). In a second study, factor structure and reliability were confirmed using a current representative sample from the German population (Altmann et al., 2019). However, the instrument and its factor structure have not yet been tested in patients with a psychological disorder. Furthermore, there is no validity study investigating the convergence of the BPEQ-12 and other attachment instruments.

### Research Questions

The presented study examined the factor structure, reliability and convergent validity of the BPEQ-12 using a clinical and a non-clinical sample of German-speaking subjects. We hypothesized a three-factor-structure with four items per scale as suggested by the authors of the short version (Altmann et al., 2018). We explore the measurement invariance regarding the presence of an anxiety disorder using two-group structural equation models. Due to the lack of research, we had no specific hypotheses. Furthermore, we investigated the convergent validity of the BPEQ-12. We hypothesized that the scales and the attachment patterns of the BPEQ-12 would correlate with the self-ratings and expert-ratings of adult attachment and that anxiety patients would be more likely to show insecure attachment assessed with the BPEQ-12 compared to healthy controls.

## Materials and Methods

### Ethics Statements

The present study is a secondary analysis. The primary study (Strauss et al., 2022) is in accordance with the guidelines for good clinical practice and was approved by the ethics committee of Friedrich-Schiller-Universität Jena, Germany (application ID: 3060-02/11). All participants gave written informed consent in accordance with the Declaration of Helsinki.

### Inclusion Criteria and Sample

In the study, patients with panic disorder (ICD-10: F41.0) and/or agoraphobia (F40.00, F40.01) as well as healthy controls were included if they had sufficient language skills and their age was between 18 and 65. Exclusion criteria for the patients were the presence of substance abuse (F1), schizophrenic disorder (F2), bipolar disorder (F31), generalized anxiety disorder (F41.1), posttraumatic stress disorder (F43.1), or personality disorder (F6). However, patients with a comorbid depression (F32, F33), social anxiety disorder (F40.1), or specific isolated phobia (F40.2) were included. Exclusion criteria for healthy controls were the presence of psychological disorder and psychotherapeutic or pharmacological treatment during the preceding 12 months. Patients and controls were matched by gender (on an individual level) and age (on the sample level). For screening the patients and the controls, we used the Structured Clinical Interview for the Diagnostic and Statistical Manual of Mental Disorders (SCID; Wittchen et al., 1997). The recruitment was carried out in the cities of Dresden and Jena, Germany. For further details regarding sample calculation and recruitment, also see the primary study (Strauss et al., 2022). In all, *N* = 174 patients and *N* = 143 healthy participants were included.

### Instruments

Besides the collection of socio-demographic data (e.g., gender, age, education, status of current relationship), several questionnaires were assessed and attachment interviews were conducted.

#### Bielefeld Partnership Expectations Questionnaire

The Bielefeld Partnership Expectations Questionnaire (BPEQ; Höger and Buschkämper, 2002; Höger et al., 2008; Pollak et al., 2008) is a self-rating of self-descriptions and expectations regarding the partner, respectively, the close relationship. It includes 30 Likert-scaled items. The reliability of the scales fear of rejection, readiness for self-disclosure, and conscious need for care is good (Cronbach’s α = 0.83–0.85; Pollak et al., 2008). Based on the individual scale values, an individual can be classified into three different attachment patterns (secure, avoidant, ambivalent) (Höger, 1999). Please note that at the time the primary study was, the short form of BPEQ had not yet been developed. In the primary study, the long version of the BPEQ was assessed. But in our secondary analysis, we considered only the 12 items of the short version of the BPEQ (see online supplement), which consists of three scales with four items each. The reliability of the scales of the short version was good (Cronbach’s α = 0.74–0.90; Altmann et al., 2019). The scales as well as the attachment patterns of the long and the short version of the BPEQ correlated highly and had good concordance, respectively (Altmann et al., 2018).

#### Experiences in Close Relationships Questionnaire

A further partner-related attachment self-rating is the Experiences in Close Relationships Questionnaire (Ehrenthal et al., 2009). With 36 Likert-scaled items it assesses attachment anxiety (ECR-ANX) and attachment avoidance (ECR-AVO) regarding feelings and behavior in close relationships. Both scales have good reliability (Cronbach’s α = 0.91/0.92; Ehrenthal et al., 2009) and convergent validity (Strauss et al., 2022).

#### Adult Attachment Prototype Rating

As an observer-rating of adult attachment, the Adult Attachment Prototype Rating (AAPR; Strauss et al., 1999), which is the German Version of the Pilkonis (1988) prototype approach, was conducted. The interview has similarities to the Adult Attachment Interview (AAI; George et al., 1996) and addresses experiences within early (parents, siblings) and current relationships (romantic partners, friends). It is a clinician-rated measure that includes seven scales: anxious, ambivalent attachment (excessive dependency, interpersonal ambivalence, and compulsive care-giving), avoidant attachment (rigid self-control, defensive separation, and emotional detachment), and secure attachment (with a single scale) (Pilkonis, 1988).

All the interviews were video-recorded. Two trained raters judged the degree of seven specific attachment prototypes (e.g., secure, excessively dependent, or emotionally detached, cf.). If the ratings by the raters differ, a consensus must be found. The reliability was acceptable (concordance of attachment prototype rated by two observers Cohen’s κ = 0.423, *p* < 0.001). In the presented study we considered the attachment styles (secure, avoidant, ambivalent) and the dimensional consensus rating of attachment security.

#### Brief Symptom Inventory

The general psychological distress due to various psychological symptoms was assessed with the Brief Symptom Inventory (BSI; Franke and Derogatis, 2000). It is the short version of the Symptom Check List (Derogatis and Cleary, 1977). The self-rating includes 53 Likert-scaled items. The resulting nine scales as well as the overall score (Global Severity Index; GSI) had good reliability (Cronbach’s α = 0.70–0.89, respectively, α = 0.96; Geisheim et al., 2002).

### Missing Data

Per item of BPEQ-12 there are up to two missing data. On the level of sum scores, we had zero to three missing data per individual. Regarding the AAPR, there were 17 subjects without observer-ratings. The MCAR-test according to Little suggested that the missing data occurred completely at random ($\chi_{\mathrm{df}=13}^{2}=19.1$, *p* = 0.119) on the scale level. We did not impute the missing values.

### Data Analysis

First, we compared the two groups regarding individual characteristics (e.g., gender, age, intensity of depression symptoms etc.) using the χ^2^-test (for categorical variables) and ANOVA (for metric variables). As effect size measures we considered Cramer’s V (> 0.1 small, > 0.3 medium, > 0.5 large effect; Cohen, 1988) and partial η^2^ (> 0.02 small, > 0.13 medium, > 0.26 large effect; Cohen, 1988).

For the validation of the factor structure we applied confirmatory factor analyses (CFA) (Lewis, 2017) on the entire sample and both groups separately. The model is specified according to Altmann et al. (2018, 2019): all three latent variables are correlated. Each latent variable loads on four items. For item number 7, 20 and 26 (the numbers refer to the long version of BPEQ) we fixed the loading on the corresponding latent variable on one. Furthermore, we used the weighted least squares mean and variance adjusted estimator (WLSMV) which is recommended for ordinal data. Considered model fit indices are the Comparative Fit Index (CFI), Tucker Lewis index (TLI), Root Mean Square Error of Approximation (RMSEA), and Standardized Root Mean Square Residual (SRMR). These fit indices are adjusted for model complexity, sensitive to model misspecifications, and cut off criteria for model selection are available (Sun, 2005). The model fit is acceptable if CFI > 0.95, TLI > 0.95, RMSEA < 0.08, and SRMR < 0.08 (Hu and Bentler, 1999; Hooper et al., 2008).

To investigate measurement invariance regarding the presence of anxiety disorder (healthy controls vs. patients), we applied several two-group models and compared their model fit indices. Unfortunately, χ^2^ difference tests are too sensitive and for alternative criteria there are no consensus (Putnick and Bornstein, 2016). When comparing two nested models, we assume measurement invariance, if ΔCFI ≤ –0.01 and ΔRMSEA ≥ 0.01. These cut-off values are conservative cut off values for metric and scalar invariance (Putnick and Bornstein, 2016). Note that metric invariance is present when the loadings are equal in both groups. Equal intercepts and loadings indicate scalar invariance. In strict measurement invariance, additionally the residual variances have to be equal.

In the next step, we considered the reliability and computed Cronbach’s α, McDonald‘s ω and average variance expected (AVE) for each scale of the entire sample and both disorder groups separately. Cronbach’s α assumes an essential tau-equivalent model (all items had the same weight) and McDonald‘s ω a tau-congeneric model (each item can have different loadings and intercepts). The AVE is the average proportion of item variance explained by factors (respectively, the latent variables in the SEM). AVE should be > 0.5.

Next, convergent validity was investigated with correlations between the BPEQ-12 scales, the ECR-R scales and the GSI as well as concordance indices (Cramer’s *V* and Cohen’s κ) between the attachment pattern of the BPEQ-12 and the attachment style of the AAPR. Correlations and Cramer’s *V* were classified according to Cohen (1988) (*r* > 0.1 small, > 0.3 medium, > 0.5 large; *V* > 0.07 small, > 0.21 medium, > 0.35 large) and Cohen’s κ according to Landis and Koch (1977) (<0 poor, > 0 slight, > 0.2 fair, > 0.4 moderate, > 0.6 substantial, and > 0.8 almost perfect agreement). Furthermore, we used a linear regression with the BPEQ-12 scales as dependent variables. The ECR-R scales and the GSI were independent variables. Last, the convergent validity of attachment pattern of the BPEQ-12 is also examined with a multi-nominal regression. The secure attachment pattern of the BPEQ-12 was the reference group.

As statistic software we used R version 4.1.0, especially the lavaan package version 0.6.10 and the semTools package version 0.5.5.

## Results

Table 1 shows the descriptive statistics for both disorder groups. Their comparison revealed that the patient sample included a higher proportion of subjects with a high-school education and more subjects with a firm relationship than the healthy controls. Hypothesis-conform, patients with panic disorder showed more symptom load (GSI), attachment avoidance (ECR-AVO), fear of rejection, and less readiness for self-disclosure and conscious need for care (BPEQ-12). Furthermore, the clinical sample included a larger proportion of individuals who classified as ambivalent attached according to the BPEQ-12, whereas the avoidant attached attachment pattern was more often present in the sample of healthy controls.

**TABLE 1:** Descriptive statistics for the disorder groups.

	Entire sample (*N* = 318)	Patients (*N* = 175)	Controls (*N* = 143)	Comparison
Female	108 (34%)	60 (34%)	49 (34%)	χ^2^(1) = 0; *V* = 0.008
Age in years	*M* = 36.2 (11)	*M* = 36 (10.5)	*M* = 36.4 (11.6)	*F*(1;316) = 0.1; η^2^ = 0.001
High school education	137 (43%)	63 (36%)	73 (51%)	χ^2^(1) = 7.3**; *V* = 0.151
Firm relationship	242 (76%)	142 (81%)	100 (70%)	χ^2^(1) = 4.9*; *V* = 0.124
Global severity index (GSI)	*M* = 0.7 (0.6)	*M* = 1.1 (0.6)	*M* = 0.3 (0.3)	*F*(1;316) = 174***; η^2^ = 0.369
Attachment anxiety (ECR)	*M* = 2.6 (1.0)	*M* = 2.7 (1.1)	*M* = 2.4 (0.9)	*F*(1;316) = 3.3^+^; η^2^ = 0.014
Attachment avoidance (ECR)	*M* = 2.1 (0.9)	*M* = 2.3 (1)	*M* = 1.9 (0.8)	*F*(1;316) = 17.8***; η^2^ = 0.055
Attachment security (AAPR)	*M* = 3.2 (0.7)	*M* = 3.0 (0.7)	*M* = 3.4 (0.7)	*F*(1;316) = 11.0***; η^2^ = 0.071
Fear of rejection (BPEQ-12)	*M* = 0.8 (0.8)	*M* = 0.9 (0.9)	*M* = 0.7 (0.7)	*F*(1;314) = 5.3*; η^2^ = 0.018
Readiness for self-disclosure (BPEQ-12)	*M* = 3 (0.9)	*M* = 2.9 (1.0)	*M* = 3.2 (0.8)	*F*(1;315) = 8.8**; η^2^ = 0.028
Conscious need for care (BPEQ-12)	*M* = 2 (0.9)	*M* = 2.2 (0.8)	*M* = 1.7 (0.8)	*F*(1;313) = 29.9***; η^2^ = 0.085

**Classification into five attachment pattern based on the values of BPEQ-12 scales**				
Secure	64 (20%)	37 (22%)	27 (19%)	
Partially-secure	134 (43%)	59 (34%)	75 (53%)	
Avoidant-withdrawing	42 (13%)	20 (12%)	22 (15%)	χ^2^(4) = 20.9***; *V* = 0.258
Ambivalent-clinging	43 (14%)	31 (18%)	12 (8%)	
Ambivalent-withdrawing	31 (10%)	25 (14%)	6 (4%)	

**Classification into three attachment pattern based on the values of BPEQ-12 scales**				
Secure	198 (63%)	96 (56%)	102 (72%)	
Ambivalent	74 (24%)	56 (33%)	18 (13%)	χ^2^(2) = 17.1***; *V* = 0.233
Avoidant	42 (13%)	20 (12%)	22 (15%)	

**Classification into three attachment styles based on AAPR**				
Secure	244 (81%)	125 (77%)	119 (86%)	
Ambivalent	22 (7%)	19 (12%)	3 (2%)	χ^2^(2) = 10**; *V* = 0.183
Avoidant	35 (12%)	19 (12%)	35 (15%)	

***p < 0.001, **p < 0.01, *p < 0.05.

The wording of items and item statistics are reported in Tables 2 and 3.

**TABLE 2:** Items of the short version of the Bielefeld Partnership Expectations Questionnaire.

Item	Item text
13	I’m afraid that my great need for attention could be too much for my partner.
17	When my partner is affectionate and loving, I sometimes doubt if he/she really means it.
20	I sometimes think that my partner would love to get rid of me.
21	I sometimes think that I love my partner more than he/she loves me.
2	It’s generally easy for me to talk to my partner about my innermost feelings.
7	It’s easy for me to talk to my partner about my feelings.
15	I can easily open up to my partner.
30	It’s fairly easy for me to tell my partner about myself: my feelings, wishes, and needs.
14	Being separated from my partner (e.g., traveling, business) makes me feel nervous and uncomfortable.
16	Separation from my partner would make my world fall apart.
22	It’s important for me that my partner thinks of me often, even when we are not together.
26	Saying good-bye is difficult for me even when separating for only a short time.

*The items were developed by Höger and Buschkämper (2002) and translated from German to English by Pollak et al. (2008). The numbering of items refers to the long version of Bielefeld Partnership Expectations Questionnaire (Höger and Buschkämper, 2002).*

**TABLE 3:** Items statistics.

	Min	Max	*M*	*SD*	Skewness	Kurtosis	*p*-value of Shapiro test
Item 13	1	5	2.006	1.161	0.972	3.008	<0.001
Item 17	1	5	1.502	0.829	1.68	5.178	<0.001
Item 20	1	5	1.617	0.93	1.634	5.348	<0.001
Item 21	1	5	1.946	1.111	1.007	3.02	<0.001
Item 02	1	5	4.107	0.994	–1.087	3.805	<0.001
Item 07	1	5	3.984	1.095	–0.925	3.008	<0.001
Item 15	1	5	4.05	1.069	–1.096	3.581	<0.001
Item 30	1	5	3.896	1.155	–0.821	2.789	<0.001
Item 14	1	5	2.58	1.262	0.334	2.06	<0.001
Item 16	1	5	3.649	1.247	–0.631	2.375	<0.001
Item 22	1	5	2.845	1.075	0.096	2.411	<0.001
Item 26	1	5	2.854	1.321	0.178	1.907	<0.001

*A p-value of Shapiro test less than 0.05 suggest that the item is not normal distributed.*

**FIGURE 1:**
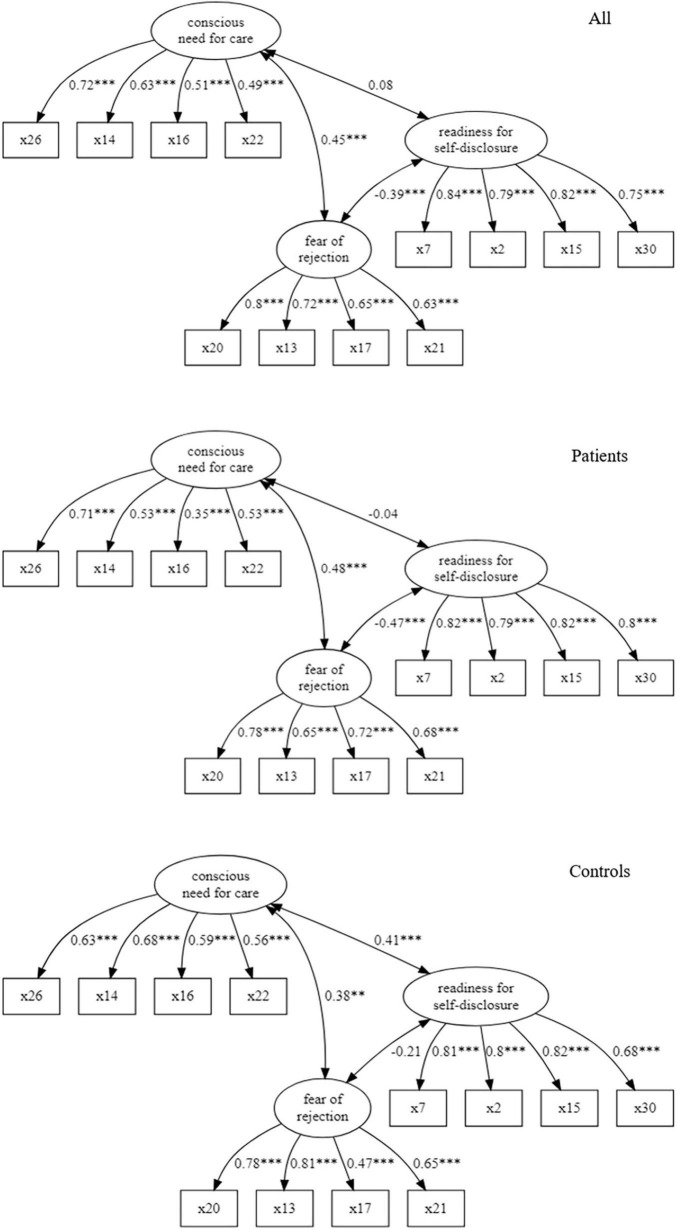
Path diagrams of three-factor model for the entire sample, patients and controls (correlations of latent variables and standardized loadings; ^***^p < 0.001, ^**^p < 0.01, *p < 0.05).

Next, we evaluated the factor structure of the BPEQ-12. The model fit of CFA was acceptable for the entire sample (CFI = 0.973, TLI = 0.966, RMSEA = 0.048, SRMR = 0.063; see Table 4; *N* = 317), the sample of patients with anxiety disorder (CFI = 0.980, TLI = 0.974, RMSEA = 0.045, SRMR = 0.072), and the sample of controls (CFI = 1, TLI = 1, RMSEA = 0, SRMR = 0.072). In Figure 1, the standardized regression coefficients and correlations of latent variables are listed for the entire sample and both groups. It should be noted that in the analysis of entire sample the standardized loading of item 22 is lower than 0.5. This holds also for item 16 in the sample of patients and item 17 in the sample of controls.

**TABLE 4:** Model fit indices when applying the SEM on the entire sample, the sample of patients and the sample of controls.

	df	Chi^2^	*p*	CFI	TLI	RMSEA	SRMR
All	51	88.0	0.0010	0.9734	0.9656	0.0481	0.0631
Patients	51	68.8	0.0488	0.9797	0.9738	0.0452	0.0723
Controls	51	51.0	0.4747	1.0000	1.0000	0.0000	0.0719

*CFI, Comparative Fit Index; TLI, Tucker Lewis index; RMSEA, Root Mean Square Error of Approximation; SRMR, Standardized Root Mean Square Residual.*

When investigating measurement invariance regarding the presence of an anxiety disorder (patients vs. healthy controls), the change of CFI and RMSEA suggest that, there is scalar, metric and strict invariance (for details see Table 5). However, subsequent analysis revealed that the loadings of item 16 and 17 differ in both groups (both *p* < 0.05, tests not reported in detail).

**TABLE 5:** Statistics of two-group models investigating various forms of measurement invariance the presence of anxiety disorder.

	Model fit							Comparison of model k and model k-1					
	df	Chi^2^	*p*	CFI	TLI	RMSEA	SRMR	df	Chi^2^	*p*	ΔCFI	ΔRMSEA	ΔSRMR
M1: configurational	102	119.8	0.1102	0.9875	0.9838	0.0334	0.0671	–	–	–	–	–	–
M2: metric	111	139.9	0.0333	0.9797	0.9758	0.0408	0.0717	9	17.9	0.0367	–0.0078	0.0074	0.0046
M3: scalar	120	159	0.0100	0.9725	0.9698	0.0456	0.0764	9	31.7	<0.001	–0.0071	0.0048	0.0047
M4: strict	132	179.4	0.0038	0.9666	0.9666	0.0480	0.0849	12	28.4	0.0049	–0.0059	0.0024	0.0084

*CFI, Comparative Fit Index; TLI, Tucker Lewis index; RMSEA, Root Mean Square Error of Approximation; SRMR, Standardized Root Mean Square Residual.*

For the scales fear of rejection and readiness for self-disclosure we found an acceptable reliability in the entire sample and both sub-samples (Cronbach’s α > 0.7; see Table 6). The reliability of the scale conscious need for care reached an acceptable level only in the sub-group of controls (α = 0.703) but not in the sub-sample of patients (α = 0.619). When quantifying the reliability with McDonalds ω, we obtained very similar results (see Table 6). The AVE was only for readiness for self-disclosure larger than 0.5 (Table 6). The scale fear of rejection narrowly missed the threshold value (but all AVE ≥ 0.489, Table 2).

**TABLE 6:** Cronbach’s α, McDonalds ω, and average variance extracted (AVE) of BPEQ-12 scales of the entire sample and both sub-groups.

	Fear of rejection	Readiness for self-disclosure	Conscious need for care
**Cronbach’s α**			
Entire sample	0.791	0.875	0.678
Patients	0.797	0.883	0.619
Controls	0.774	0.853	0.703

**McDonalds ω**			
Entire sample	0.785	0.875	0.688
Patients	0.781	0.881	0.616
Controls	0.765	0.856	0.715

**AVE**			
Entire sample	0.494	0.638	0.367
Patients	0.489	0.655	0.314
Controls	0.497	0.597	0.381

The evaluation of convergent validity of the BPEQ-12 scales revealed that fear of rejection is mainly associated with higher attachment anxiety (assessed with the ECR-R) and readiness for self-disclosure mainly with lower attachment avoidance. This holds for both the correlation and the regression analysis (large effect sizes, see Tables 7, 8). In the regression analysis, the scale conscious need for care was associated with higher ECR-ANX, higher GSI, and lower ECR-AVO. The corresponding effect sizes were small.

**TABLE 7:** Correlations of BPEQ-12 scales and other scales (*N* = 314; ECR-ANX attachment anxiety, ECR-AVO attachment avoidance, GSI Global Symptom Index).

	Fear of rejection	Readiness for self-disclosure	Conscious need for care
Attachment anxiety (ECR)	0.709***	−0.286***	0.301***
Attachment avoidance (ECR)	0.384***	−0.723***	–0.052
Attachment security (AAPR)	−0.29***	0.337***	–0.091
Global Severity Index (GSI)	0.42***	−0.268***	0.37***

***p < 0.001, **p < 0.01, *p < 0.05.

**TABLE 8:** Prediction of BPEQ-12 scales using multi-variate regression.

	Fear of rejection			Readiness for self-disclosure			Conscious need for care		
	*b*	*SE*	η^2^	*b*	*SE*	η^2^	*b*	*SE*	η^2^
intercept	−0.5*	(0.22)	0.02	3.93***	(0.25)	0.45	1.5***	(0.3)	0.08
ECR-ANX	0.49***	(0.04)	0.37	0.11**	(0.04)	0.02	0.29***	(0.05)	0.1
ECR-AVO	–0.01	(0.04)	0	−0.73***	(0.05)	0.45	−0.33***	(0.06)	0.1
AAPR-SEC	–0.04	(0.05)	0	0.11*	(0.06)	0.01	0.02	(0.07)	0
GSI	0.2***	(0.06)	0.04	–0.03	(0.06)	0	0.52***	(0.08)	0.13

ECR-ANX attachment anxiety assessed with ECR, ECR-AVO attachment avoidance assessed with ECR, AAPR-SEC attachment security assessed with AAPR, GSI Global Severity Index. ***p < 0.001, **p < 0.01, *p < 0.05, ^+^p < 0.1; N = 314.

**FIGURE 2:**
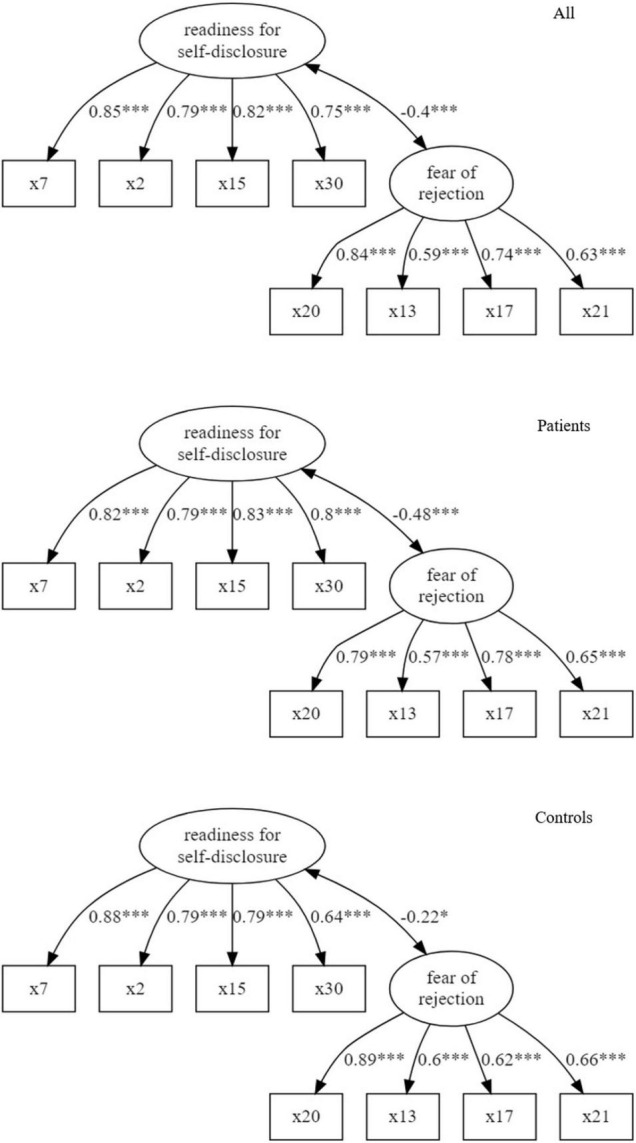
Path diagrams of two-factor model for the entire sample, patients and controls (correlations of latent variables and standardized loadings; ^***^p < 0.001, ^**^p < 0.01, *p < 0.05).

The results reported above suggested that only two scales are reliable and valid. Therefore, we evaluated in a *post-hoc* analysis a CFA including only fear of rejection and readiness for self-disclosure. All four fit indices were acceptable in the entire sample (CFI = 0.996, TLI = 0.994, RMSEA = 0.027, SRMR = 0.054), the patient group (CFI = 0.997, TLI = 0.996, RMSEA = 0.025, SRMR = 0.065) and control group (CFI = 1, TLI = 1, RMSEA = 0, SRMR = 0.0495). The loadings of two factor model are shown in Figure 2.

Regarding the attachment patterns of the BPEQ-12, we first compared the observed distribution of the five attachment patterns with distributions reported by Höger et al. (2008) and Altmann et al. (2018). Neither the patient sample nor the control sample had a distribution similar to these studies (all *p* < 0.001). However, we found that anxiety patients had insecure attachment more frequently than healthy controls (medium effect sizes). This holds for both, a categorization into five and three attachment patterns assessed with the BPEQ-12 (see Table 1). Furthermore, we found a significant but slight convergence between the attachment pattern of the BPEQ-12 and the attachment styles of the AAPR (for the three pattern secure, avoidant, and ambivalent: χ^2^(4) = 31.2^***^, *V* = 0.229, κ = 0.174, *N* = 298; two patterns secure and insecure: χ^2^(1) = 12.9^***^, *V* = 0.208, κ = 0.187, *N* = 298). Multi-nominal regression revealed that the ambivalent attachment pattern of the BPEQ-12 is mainly predicted by the ECR-ANX and GSI, whereas the avoidant attachment pattern is mainly associated with the ECR-AVO (see Table 9). Attachment security assessed by AAPR was neither predictive for the ambivalent pattern nor for the avoidant pattern.

**TABLE 9:** Prediction of attachment pattern of BPEQ-12 using multi-nominal regression.

	Ambivalent vs. secure			Avoidant vs. secure		
	*b*	*SE*	exp(*b)*	*b*	*SE*	exp(*b*)
Intercept	−5.76***	(1.33)		−7.9***	(1.62)	
ECR-ANX	1.31***	(0.22)	3.69	0.31	(0.26)	1.36
ECR-AVO	0.61*	(0.26)	1.85	2***	(0.3)	7.41
AAPR-SEC	–0.28	(0.28)	0.75	0.35	(0.34)	1.42
GSI	0.93**	(0.31)	2.54	–0.23	(0.39)	0.79

*ECR-ANX, attachment anxiety assessed with ECR-R; ECR-AVO, attachment avoidance assessed with ECR-R; AAPR-SEC, attachment security assessed with AAPR; GSI, Global Severity Index assessed with BSI.*

***p < 0.001, **p < 0.01, *p < 0.05, ^+^p < 0.1; N = 313.

Due to the low convergence between the attachment styles of the AAPR and the BPEQ-12, we conducted the following *post hoc* analyses. First, we tried to replicate sample partitioning into several attachment patterns proposed by Höger and Buschkämper (2002). For this we applied a latent class analysis (LCA) on the three BPEQ-12 scales. In concordance with Höger and Buschkämper (2002), the Bayes Information criteria, the entropy value, and the parametric bootstrapped likelihood ratio test suggested a solution with five latent classes (respectively, five attachment pattern). However, the concordance of these “new” attachment patterns with the “original” five attachment patterns was weak according to Cohen’s κ [χ^2^(16) = 291.8^***^, *V* = 0.482, κ = 0.146, *N* = 314]. Then, we considered a solution of LCA with three latent classes. These three attachment patterns correlated moderately with the “original” BPEQ-12 attachment patterns [χ^2^(4) = 196.2^***^, *V* = 0.559, κ = 0.488, *N* = 314]. In other words, our partitioning of the data confirms the three patterns by Höger and Buschkämper (2002). However, the concordance between these new BPEQ-12 attachment patterns and the three AAPR attachment styles was weak [χ^2^(4) = 19.5^**^, *V* = 0.18, κ = 0.109, *N* = 314].

In a further *post hoc* analysis, we determined “new” attachment patterns of the BPEQ-12 using AAPR attachment styles as reference instead of the data-driven method above. We randomly divided the sample into two halves: a learning sample and an evaluation sample. Neither sex, age, high-school education, firm relationship nor the presence of anxiety was able to predict the assignment to the random groups. Based on the learning sample, we computed the averages of the BPEQ-12 scales depending on the AAPR attachment styles. Then, we classified each individual in the evaluation sample according to the smallest distance to these three “cluster centroids.” According to Cohen’s κ, the concordance between the resulting BPEQ-12 attachment pattern and the “original” attachment style of AAPR was significant, but slight [learning sample: χ^2^(6) = 15.3^**^, *V* = 0.225, κ = 0.157; evaluation sample B: χ^2^(6) = 17.9^**^, *V* = 0.243, κ = 0.162].

## Discussion

The present study evaluated the factor structure and convergent validity of the short version of the Bielefeld Partnership Expectations Questionnaire (BPEQ-12) in patients with anxiety disorder and healthy controls. The confirmatory factor analysis confirmed the expected structure with three factors and four items per factor in the entire sample, the sample of anxiety patients and the sample of healthy controls. Investigations of measurement invariance suggest strict measurement invariance. However, the loadings of item 16 and 17 are different in both groups.

The reliability was acceptable (Cronbach’s α > 0.7) for all three scales in our sub-sample of healthy controls. Our findings correspond to Höger and Buschkämper (2002), Kirchmann et al. (2007), Petrowski et al. (2010), and Altmann et al. (2018, 2019) (Cronbach’s α = 0.72-0.95), who examined only healthy individuals. However, in our sub-sample of anxiety patients, the scales fear of rejection and readiness for selfdisclosure had good reliability, whereas the scale conscious need for care missed the threshold for acceptable reliability (α < 0.7). Interestingly, in the studies mentioned above, the scale conscious need for care showed the lowest values for Cronbach’s α compared to the other scales. A reason might be that three items of this scale had a comparatively low (standardized) loading in the sub-sample of anxiety patients. Accordingly, in future studies, the scale conscious need for care should be used with caution, especially when anxiety patients are examined.

The convergent validity of the BPEQ-12 scales was examined with correlation and regression analyses. Our results suggest that, despite the fact that the BPEQ-12 measure three dimensions, the BPEQ-12 ought rather be assigned to two-dimensional models (e.g., Bartholomew and Horowitz, 1991; Brennan et al., 1998) than to three-dimensional models (e.g., Bäckström and Holmes, 2007). The former models include the dimensions attachment anxiety and avoidance and derive attachment security based on the constellation of both dimensions. In contrast, the model of Bäckström and Holmes (2007) measure attachment anxiety, avoidance, and security directly. Our results from the regression analysis showed that attachment security assessed with AAPR is not associated with any of the BPEQ-12 scales, whereas fear of rejection is related to attachment anxiety of ECR-R and readiness for self-disclosure to attachment avoidance of ECR-R. The lack of convergent validity of the scale conscious need for care corresponds to findings by Grau et al. (2003) who examined the original version of BPEQ. All in all, the low reliability and low convergent validity suggests the exclusion of the scale conscious need for care. However, we cannot rule out that sufficient reliability will be found in other clinical samples. Also, it is possible that convergence might be found with other attachment measures not considered in the present study. For the computation of attachment patterns according to Höger and Buschkämper (2002), all three scales must be assessed.

Furthermore, our results suggest discriminant validity. We found medium correlations (*r* > 0.3) between the symptom load measured with BSI and the three attachment scales of BPEQ-12. This means that the BPEQ-12 measures not the distress due to various psychological symptoms, but a related concept.

Furthermore, we evaluated the classification into the attachment patterns according to Höger and Buschkämper (2002), Grau et al. (2003), and Höger et al. (2008). Indeed, our regression analysis revealed that the avoidant attachment pattern of the BPEQ-12 is mainly predicted by the ECR-R scale attachment avoidance and that the ambivalent attachment pattern of the BPEQ-12 is associated with the ECR-R scale attachment anxiety. This corresponds with Grau et al. (2003). Moreover, the resulting patterns of an LCA with three latent classes correspond to the three patterns (secure, avoidant, ambivalent) classified according to Höger et al. (2008). However, the fit indices of our LCA indicate a solution with five patterns which correspond only slightly with the “original” five patterns of Höger et al. (2008) (see first *post hoc* analysis).

A critical point is the low concordance between the attachment patterns of the BPEQ-12 and the attachment styles of the AAPR. This low concordance is also present for the BPEQ-12 attachment pattern classification that was developed based on the attachment styles of the AAPR (see second *post hoc* analysis). In contrast, we found evidence for the convergent validity of the BPEQ-12 and ECR-R scales. These findings support the assumption by Bartholomew and Shaver (1998): the convergent validity is higher when the method of both considered attachment instruments matches (BPEQ-12 and ECR-R are self-ratings) and lower when the method does not match (the AAPR is an interview-based expert-rating).

A general point to discuss is whether there exist only the three attachment patterns secure, ambivalent, and avoidant or whether mixed patterns may also be possible (e.g., partially secure in the model of Höger et al., 2008). The attachment concepts behind the BPEQ (Höger et al., 2008) and the AAPR (Strauss et al., 1999), for example, assume that an individual can have secure and insecure “parts.” Both instruments measure attachment-relevant dimensions in the first step. In the AAPR, experts rate the degree of presence of seven prototypes on the basis of video-recorded interviews. In the BPEQ, the subjects themselves rate the degree of the three attachment dimensions. In a second step, the dimensions are mapped into a small set of attachment patterns (resp. styles) using an instrument-specific algorithm. When two attachment dimensions have a similar intensity (e.g., attachment security and attachment avoidance), the subject can be assigned to different attachment patterns depending on which attachment dimension is slightly more present. The BPEQ (Höger et al., 2008) avoids such difficult to decide classifications with additional mixed patterns (e.g., partially secure). However, the simultaneous presence of secure and insecure “parts” may be one reason why dimensional models of attachment are better suited for measuring individual differences (Fraley and Roisman, 2014; Shi et al., 2014; Fraley et al., 2015; Jewell et al., 2019). The classification in a small subset of attachment pattern (or styles) based on attachment dimensions include a loss of information. However, in other contexts, categorical models are more suited, e.g., to describing different types of patients and their attachment-related behavior in the context of medical care (Strauss and Brenk-Franz, 2016). The benefit of the BPEQ-12 is that the instrument provides attachment dimensions and categories.

## Limitations

One of the limitations is the focus on one specific disorder group (panic disorder and/or agoraphobia). Also, the clinical sample was not representative. Furthermore, the appropriateness of the AAPR as reference is limited by its moderate inter-rater-reliability and the untypical distribution of the attachment styles of this instrument. Moreover, it should be noted that, similar to Altmann et al. (2018), the long version of the BPEQ was assessed and that the analyses were thus based on 12 items selected from the long version. Last, the sample size of both groups was below *N* = 250 which leads to biased model fit indices (especially TLI and RMSEA) (Hu and Bentler, 1999).

## Conclusion

The BPEQ-12 is suitable to measure partner-related adult attachment. However, only the reliability of the scales fear of rejection and readiness for self-disclosure was acceptable. The former showed convergent validity with higher attachment anxiety and the latter with lower attachment avoidance assessed with the ECR-R. The scale conscious need for care should be used with caution. It missed the threshold for acceptable reliability and had low convergent validity regarding attachment anxiety, attachment avoidance, and attachment security. Despite the measurement of three scales, the BPEQ-12 can be assigned to the two-dimensional attachment models including attachment-avoidance and attachment-anxiety.

## Data Availability Statement

The datasets presented in this article are not readily available because they are still used for further analyses. The data that support the findings of this study are available from the senior authors, BS and KP, upon reasonable request. Requests to access the datasets should be directed to BS, bernhard.strauss@med.uni-jena.de.

## Ethics Statement

The studies involving human participants were reviewed and approved by Committee of Jena University Hospital, Jena, Germany (ID 3060-02/11). The patients/participants provided their written informed consent to participate in this study.

## Author Contributions

UA conducted the data analysis and did the first drafting. KB-F contributed with literature research. BS contributed with statistical advice. KP conceptualized the design of the research and provided helpful advice for drafting and statistical analysis. All authors contributed to the article and approved the submitted version.

## Conflict of Interest

The authors declare that the research was conducted in the absence of any commercial or financial relationships that could be construed as a potential conflict of interest.

## Publisher’s Note

All claims expressed in this article are solely those of the authors and do not necessarily represent those of their affiliated organizations, or those of the publisher, the editors and the reviewers. Any product that may be evaluated in this article, or claim that may be made by its manufacturer, is not guaranteed or endorsed by the publisher.
